# Human or animal? Finding the best ultrasound soft tissue model for foreign body evaluation

**DOI:** 10.1186/2036-7902-4-S1-A18

**Published:** 2012-12-18

**Authors:** Daniel Jafari, KJ Cody, AJ Dean, FS Shofer, KY Jenq, JI Fischer, NL Panebianco

**Affiliations:** 1 Department of Emergency Medicine, Hospital of the University of Pennsylvania, Philadelphia, PA, USA; 2 Department of Emergency Medicine, Pennsylvania Hospital, Philadelphia, PA, USA; 3 Department of Emergency Medicine, Thomas Jefferson University Hospital, Philadelphia, PA, USA

## Background

A variety of animal models have been used for research into the sonographic evaluation of soft tissue foreign bodies. The wide range of reported accuracies in FB detection in these studies may be due to some tissue models being easier to image than human tissue.

## Objective

To determine which among commonly used animal tissue models for FB detection most closely approximates human soft tissue.

## Methods

99 sonographic images (59 still, 40 video clips) of soft tissues from healthy humans, chicken breasts and thighs, turkey thighs, beef chops and pork feet were obtained using a high frequency linear transducer. Video clips were 6 seconds and the scanning depth was 1.5 to 3.3 cm. Clips and images were grouped separately in random order, and consisted of hand (4 stills, 6 clips), arm (3, 2), foot (6, 3), leg (4, 2), flank (2, 4), chicken breasts (5, 5), chicken thighs (7, 2), turkey thighs (8, 6), beef chops (8, 3), and pork feet (13, 7). 7 experienced ED sonologists reviewed images and rated as “human tissue”, “non-human tissue”, or “don’t know”. Responses were converted into a binary variable, and raw percentages per sonologist and per animal type were calculated. To determine which animal tissue was most frequently identified as human, logistic regression was used clustering on sonologist.

## Results

Correct identification rate was 67% for the trunk, 57% for the hand and leg, 37% for the arm, and 17% for the foot. For animal tissue models, the rate of identification as human tissue was 61% for the pork feet, 35% for the chicken thighs, 26% for the beef chops, 23% for the turkey thighs, and 13% for the chicken breasts. Pork feet were the most likely animal tissue to be identified as human tissue (p<0.0001, OR=1.9, 95% CI 1.2-3.0), and chicken breasts were the least (p<0.0001, OR=0.16, 95% CI 0.07-0.35).

## Conclusion

Among animal tissues, pork feet most closely approximate human tissues. Experienced sonologists performed poorly in distinguishing human from animal tissue. Educational programs and future studies might be optimized by the use of this animal soft tissue model.

